# 3D-printed porous titanium versus polyetheretherketone cages in lateral lumbar interbody fusion: a systematic review and meta-analysis of subsidence

**DOI:** 10.3389/fmed.2024.1389533

**Published:** 2024-12-18

**Authors:** Shu-Xin Liu, Teng-Hui Zeng, Chien-Min Chen, Li-Ru He, An-Ping Feng, Shang-Wun Jhang, Guang-Xun Lin

**Affiliations:** ^1^Department of Orthopedics, Panjin Central Hospital, Panjin, Liaoning, China; ^2^Department of Orthopedics, The Second People’s Hospital of Shenzhen (The First Affiliated Hospital of Shenzhen University), Shenzhen, Guangdong, China; ^3^Division of Neurosurgery, Department of Surgery, Changhua Christian Hospital, Changhua, Taiwan; ^4^Department of Leisure Industry Management, National Chin-Yi University of Technology, Taichung, Taiwan; ^5^Department of Biomedical Sciences, National Chung Cheng University, Chiayi, Taiwan; ^6^Department of Anesthesia and Surgery, The First Affiliated Hospital of Xiamen University, Xiamen University, Xiamen, Fujian, China; ^7^Department of Orthopedics and Traumatology of Traditional Chinese Medicine, The Third Hospital of Xiamen, Xiamen, China; ^8^Department of Orthopedics, The First Affiliated Hospital of Xiamen University, School of Medicine, Xiamen University, Xiamen, China; ^9^The School of Clinical Medicine, Fujian Medical University, Fuzhou, Fujian, China

**Keywords:** lateral lumbar interbody fusion, polyetheretherketone, interbody cage, titanium, subsidence

## Abstract

**Background:**

Cage subsidence frequently complicates lumbar fusion procedures, including lateral lumbar interbody fusion (LLIF), potentially leading to recurrent pain, impaired fusion, and accelerated degeneration of adjacent segments. A critical factor influencing cage subsidence is the selection of material. Polyetheretherketone (PEEK) and three-dimensional printed titanium (3D-Ti) cages are commonly used in LLIF procedures, each offering distinct advantages. However, these materials possess inherent property differences that may translate into divergent settling rates. To contribute to this discourse and offer insights, this systematic review and meta-analysis aims to compare the rates of cage subsidence between 3D-Ti and PEEK cages in LLIF.

**Methods:**

A meticulous systematic search that employs distinct MeSH terms was conducted in major electronic databases (MEDLINE, PubMed, Embase, Scopus, Web of Science, and Cochrane) up to December 20, 2023. The quality of inclusion was measured using the Newcastle-Ottawa Scale (NOS) for non-randomized trials. The primary outcome measure was cage subsidence, while the secondary outcome involved evaluating subsidence within each treatment segment using the Marchi classification.

**Results:**

The review included 265 patients (441 segments) from three studies. All with NOS ratings exceeding 5 stars. In the analysis, 189 segments (42.9%) underwent LLIF with 3D-Ti cages, while 252 segments (57.1%) participated in LLIF with PEEK cages. Overall, the cage subsidence rate was significantly lower with 3D-Ti compared to PEEK (*p* < 0.00001, OR = 0.25; 95% CI 0.14 to 0.44). Specifically, the 3D-Ti group exhibited a markedly lower subsidence rate, categorized by grade I, II, and III, compared to the PEEK group (*p* < 0.05). Furthermore, the incidence of severe subsidence was significantly reduced in the 3D-Ti group compared to the PEEK group (*p* = 0.0004, OR = 0.17; 95% CI 0.07 to 0.46).

**Conclusion:**

The study concludes that the subsidence rate associated with 3D-Ti cages in LLIF is notably lower than that observed with PEEK cages, underscoring the potential advantages of 3D-Ti cages in mitigating cage subsidence.

## 1 Introduction

Lateral lumbar interbody fusion (LLIF) is a surgical procedure designed to address various spinal degenerative conditions by accessing the lumbar spine from a lateral or side approach (1, 2). This approach allows the insertion of an intervertebral fusion device into the disc space, promoting fusion between adjacent vertebrae (3). LLIF has gained popularity as a minimally invasive alternative to traditional posterior approaches, offering advantages such as reduced muscle dissection and decreased disruption of posterior spinal structures (4, 5).

A key advantage of LLIF is its ability to restore spinal alignment through the use of large interbody cages. The insertion of a large cage in LLIF can help restore the height of the intervertebral space, improve biomechanical strength, and promote interbody fusion (6). However, despite these benefits, the use of cages introduces specific challenges, most notably the risk of cage subsidence. The incidence of cage subsidence after LLIF has been reported to range from 3.3 to 39.6%, depending on factors such as the cage material, surgical approach (which contains the factor of cage’s design and size), and evaluation tools (7, 8). When cage subsidence occurs, it compromises the mechanical stability of the spine and places additional stress on neighboring segments, potentially accelerating their degeneration (9). This can lead to a cascade of clinical problems, including recurrent pain, neurological deficits, and the need for revision surgery (10).

The material composition of interbody cages plays a crucial role in determining the risk of subsidence and overall surgical outcomes. Common causes of subsidence include inadequate fixation with a nail bar system, inappropriate selection of the fusion device, endplate loss, and underlying patient factors such as osteoporosis (11–13). The choice of cage material, particularly between polyetheretherketone (PEEK) and three-dimensionally printed titanium (3D-Ti), is of particular interest in modern spine surgery (14). PEEK has a modulus of elasticity similar to cortical bone, minimizing stress shielding and encouraging more natural load distribution (15). In contrast, titanium offers greater initial stability but may lead to stress shielding due to its higher modulus of elasticity (16). In addition, 3D-Ti cages that the porous structure of the 3D-Ti cage also enables the cage to have an appropriate structural stiffness which strongly affects the possibility of subsidence. PEEK’s radiolucency enables clearer postoperative imaging, whereas titanium’s radiopacity can obscure imaging, complicating the assessment of fusion progress (17). However, 3D-Ti cages excel in promoting osseointegration due to their porous structure, which fosters bone growth and enhances the biological interface between the cage and the vertebrae (18). While PEEK is biocompatible, it does not inherently promote bone growth without additional coating with osteoconductive materials (19).

The aim of this paper is to systematically compare the subsidence rates of 3D-Ti and PEEK cages in LLIF procedures. By providing evidence-based insights, this study seeks to guide surgeons in selecting the most appropriate cage material for their patients, representing the first meta-analysis to compare these two materials specifically in the context of LLIF applications.

## 2 Methods and materials

### 2.1 Study strategy

Adhering rigorously to the Preferred Reporting Items for Systematic Reviews and Meta-Analyses (PRISMA) guidelines, we performed a systematic and comprehensive search across prominent academic databases (20). This exhaustive search database included MEDLINE, PubMed, Embase, Scopus, Web of Science, and Cochrane. The overarching objective of this methodologically stringent inquiry was to undertake a comparative analysis of the incidence of cage subsidence following the utilization of 3D-Ti and PEEK cages in the context of LLIF. Conducted on December 20, 2023, the selected keywords, namely “lateral lumbar interbody fusion,” “direct lumbar interbody fusion,” “extreme lumbar interbody fusion,” “eXtreme lumbar interbody fusion,” “LLIF,” “XLIF,” “ELIF,” “DLIF,” “three-dimensional printed,” “3D-printed,” “porous,” “titanium,” “polyetheretherketone,” and “subsidence,” were thoughtfully curated to enable a thorough and targeted exploration of the extant literature on the subject.

To improve the completeness of the search strategy, a secondary review of the references cited in the selected articles was carried out to further improve the breadth and depth of the literature review (21).

### 2.2 Inclusion and exclusion criteria

#### 2.2.1 Inclusion criteria

(1) Age greater than 18 years, a diagnosis of degenerative lumbar disease or adult spinal deformity, and undergoing treatment with LLIF surgery. (2) Studies explicitly comparing cage subsidence between 3D-Ti and PEEK cages following LLIF. (3) Original research articles including randomized controlled trials (RCTs), cohort studies, and case–control studies. (4) Articles published in English.

#### 2.2.2 Exclusion criteria

(1) Revision surgery, patients with indications of infection and tumor. (2) Reviews, letters, editorials, and meeting abstracts. (3) A study of patients who have undergone transforaminal lumbar interbody fusion or the use of common titanium cages. (4) Papers in languages other than English.

### 2.3 Data extraction

The responsibility for the meticulous screening of all articles derived from the systematic search was distributed among two designated authors. In instances where conflicts emerged during the screening process, a judicious resolution was sought through consultation with a third co-author (22). Discrepancies were systematically addressed through collaborative discussions aimed at achieving a consensus reflecting the collective expertise within the research team.

Throughout the screening process, due diligence was applied in the evaluation of titles and abstracts to determine their relevance to the parameters defined in the study. Instances where the information provided in the titles and abstracts was ambiguous or insufficient prompted a thorough review of the entire article. This rigorous methodological approach was deliberately employed to assess study eligibility in alignment with pre-established inclusion and exclusion criteria.

The primary outcome was the subsidence of the cage. The secondary outcome was the measurement of cage subsidence in each treatment segment using the Marchi grade classification. According to the Marchi et al. (23) grading classification, the categorization of cage subsidence is delineated as follows: Grade 0 denotes a postoperative disc height loss ranging from 0 to 24%; Grade I means a subsidence within the range of 25 to 49%; Grade II encompasses a subsidence extent of 50 to 74%; and Grade III designates a subsidence involving a 75 to 100% loss of postoperative disc height. Subsidence was classified as a binary outcome, differentiating between the absence of subsidence (Marchi grade 0) and the presence of subsidence (Marchi grades I, II, or III). Furthermore, severe subsidence was specifically defined by the presence of Grade II or III, highlighting instances of more pronounced reduction in disc height.

### 2.4 Quality assessment and publication bias

The evaluation of the methodological rigor of studies incorporated into this meta-analysis used the Newcastle-Ottawa Scale (NOS) for non-randomized studies (24). The NOS scale evaluates the quality of studies by assessing key parameters, including selection, comparability, and outcome. Studies that achieve or exceed a predetermined threshold of five “stars” are deemed to meet high-quality criteria based on the specified rating criteria. The credibility of the evidence was assessed using the Grading of Recommendations Assessment, Development and Evaluation (GRADE) evaluation method. This evaluation considers several factors, including the risk of publication bias, the precision of the results, and the magnitude of the effects of treatment. The resulting quality of the evidence is then stratified into four hierarchical grades: high, moderate, low, and very low.

### 2.5 Statistical analysis

To synthesize the available evidence, we conducted rigorous statistical meta-analyses using Review Manager 5.3 software. For continuous data, we calculated weighted mean differences (WMD) with 95% confidence intervals (CI). Dichotomous outcomes were expressed as odds ratios (OR) with the corresponding 95% CI. Heterogeneity was quantified using the I^2^ statistic, with a threshold of I^2^ ≥ 50% indicating substantial heterogeneity. In the absence of significant statistical heterogeneity (*p* > 0.1, I^2^ < 50%), we employed a fixed-effects model for pooling. However, in the presence of significant heterogeneity (*p* < 0.1, I^2^ ≥ 50%), we utilized a random-effects model. Statistical significance was established at *p* < 0.05.

To assess possible publication bias, we incorporated funnel plots into the analysis. These visual tools facilitated the detection of asymmetry, providing information on the possible influence of publication bias on the observed results.

## 3 Results

### 3.1 Search results and study characteristics

**Figure 1:**
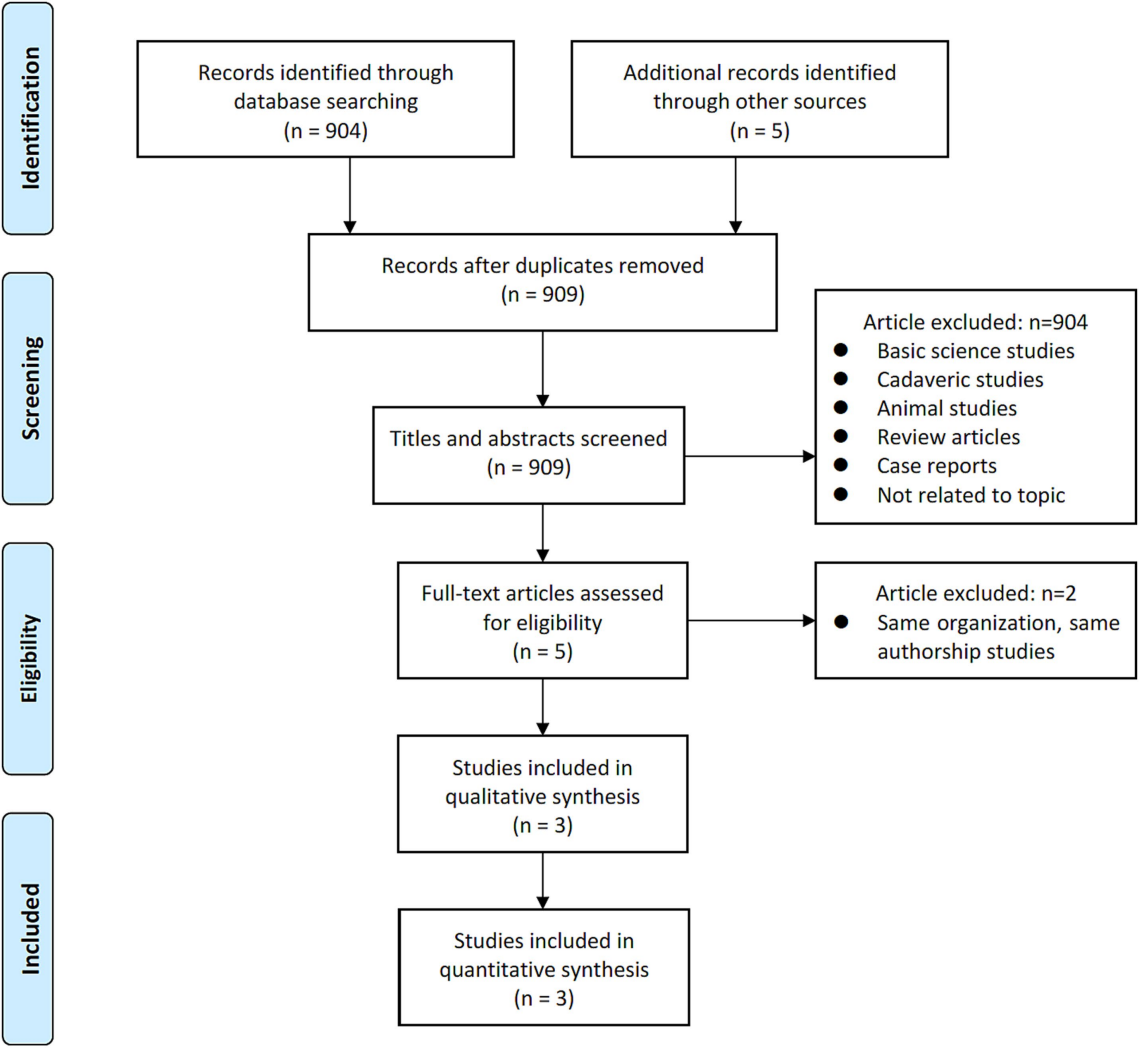
Flowchart of study selection for meta-analysis.

A comprehensive and systematic search of the literature yielded three studies (Figure 1) that met the predetermined inclusion criteria. In particular, all three studies included in this analysis adopted a retrospective design. Collectively, these studies encompassed a cohort of 265 patients (441 levels), with 189 levels assigned to the 3D-Ti group and 252 levels assigned to the PEEK group (25–27). Geographically, two studies originated in the United States, while the remaining one originated in Japan. The most operated segments were L4–5. An analysis of mean age revealed a value of 66.07 years in the 3D-Ti group and 66.27 years in the PEEK group (Table 1).

**Table 1:** Characteristics of the included studies.

Study (Year)	Study Design	Country	Group	Gender (M/F)	Mean age (range), years	Operation level	Fused levels	BMI	BMD	Follow-up
Adl Amini (2021) (25)	Retrospective	USA	3D-Ti	27/11	59.5 ± 9.5	L1–2 (5); L2–3 (11); L3–4 (20); L4–5 (31)	1 (21); 2 (8); 3 or more (9)	26.3 ± 2.75	127.8 ± 17.85 (mg/cm^3^)	12 months
			PEEK	35/40	60 ± 9.5	L1–2 (2); L2–3 (11); L3–4 (46); L4–5(60)	1 (36); 2 (34); 3 or more (5)	27.2 ± 3.9	127.2 ± 31.7 (mg/cm^3^)	
Alan (2023) (26)	Retrospective	USA	3D-Ti	NR	64 ± 9.2	NR	1 (56); 2 (16); 3 (16); 4 (9)	NR	NR	12 months
			PEEK		65 ± 7.9		1 (48); 2 (22); 3 (21); 4 (6)			
Segi (2023) (27)	Retrospective	Japan	3D-Ti	5/6	74.7 ± 6.3	L2–3 (5); L3–4 (10); L4–5(10)	1 (1); 2 (6); 3 (4)	NR	0.692 ± 0.088 (g/cm^2^)	3 months
			PEEK	5/16	73.8 ± 7.1	L1–2 (2); L2–3 (14); L3–4 (13); L4–5(9)	1 (2); 2 (10); 3 (8); 4 (1)		0.693 ± 0.122 (g/cm^2^)	

3D-Ti, 3D-printed porous titanium; PEEK, polyetheretherketone; NR, not report; BMI, body mass index; BMD, bone mineral density.

Adl Amini et al. (25) employed a standalone LLIF approach in their investigation, while in the study by Alan et al. (26), approximately one third of the patients underwent posterior instrumentation in conjunction with LLIF, in addition to Segi et al. (27), further differed in their surgical approach, reporting the use of posterior instrumentation along with LLIF (Table 2).

**Table 2:** Related data on characteristics of the cages.

Study (Year)	Group	Model (manufacturer)	Posterior instrumentation	Cage filling material
Adl Amini (2021) (25)	3D-Ti	Modulus XLIF (NuVasive); Lateral Spine Truss System (4WEB Medical)	NR	Either recombinant human bone morphogenetic protein-2 (rhBMP2), demineralized allograft fibers or both
	PEEK	Cougar (DePuy Synthes); XLIF (NuVasive)	NR	
Alan (2023) (26)	3D-Ti	NR	33/97	Demineralized bone matrix
	PEEK		32/97	
Segi (2023) (27)	3D-Ti	Modulus XLIF (NuVasive)	All cases	Custom-made hydroxyapatite mass
	PEEK	sPEEK (NuVasive)	All cases	Grafting bone

3D-Ti, 3D-printed porous titanium; PEEK, polyetheretherketone; NR, not report.

**Figure 2:**
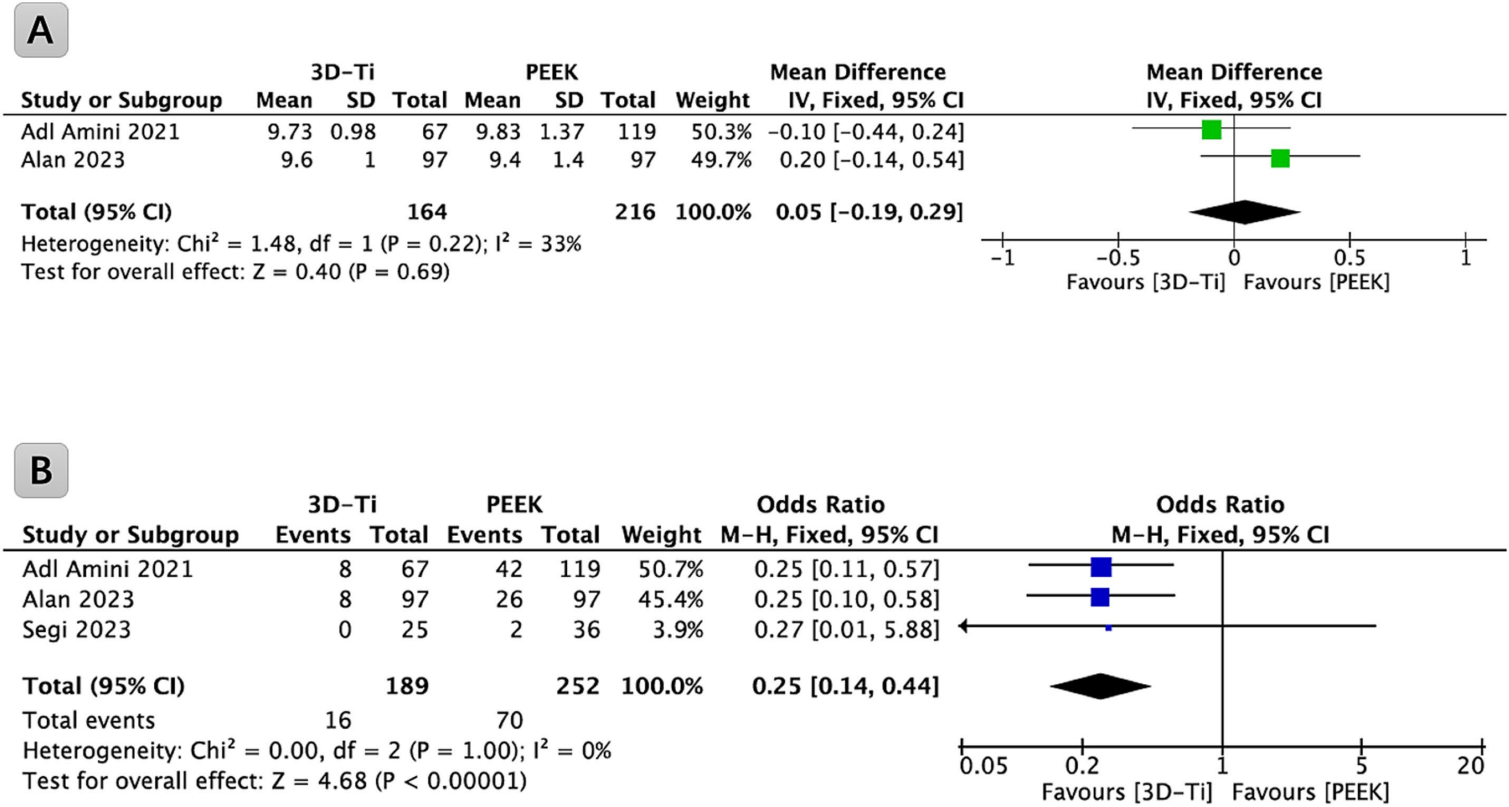
**(A)** Forest plot comparing cage height in LLIF between 3D-Ti group and PEEK group; **(B)** Forest plot comparing cage subsidence in LLIF between 3D-Ti group and PEEK group. LLIF, lumbar lateral interbody fusion, 3D-Ti, 3D-printed porous titanium, PEEK, polyetheretherketone.

Two papers contributed data related to the cage height. Upon comprehensive analysis, the results indicated a lack of statistically significant differences in cage height between the 3D-Ti cage group and the PEEK group (*p* = 0.69, WMD = 0.05; 95% CI: −0.19 to 0.29; Figure 2A).

### 3.2 Cage subsidence

Among the identified studies, three provided relevant data on the subsidence of the cage and were incorporated into the analysis (Figure 2B). Collectively, these studies encompassed a cohort of 265 patients (441 levels) undergoing LLIF surgery. Statistical analysis revealed a significantly lower incidence of cage subsidence in the 3D-Ti cage group compared to the PEEK cage group (*p* < 0.00001, OR = 0.25; 95% CI: 0.14 to 0.44). Specifically, the subsidence rate of the cage (Marchi grades I, II, and III) in the 3D-Ti group was 8.47%, while the PEEK group exhibited a subsidence rate of 27.78%.

### 3.3 Marchi grade classification

**Figure 3:**
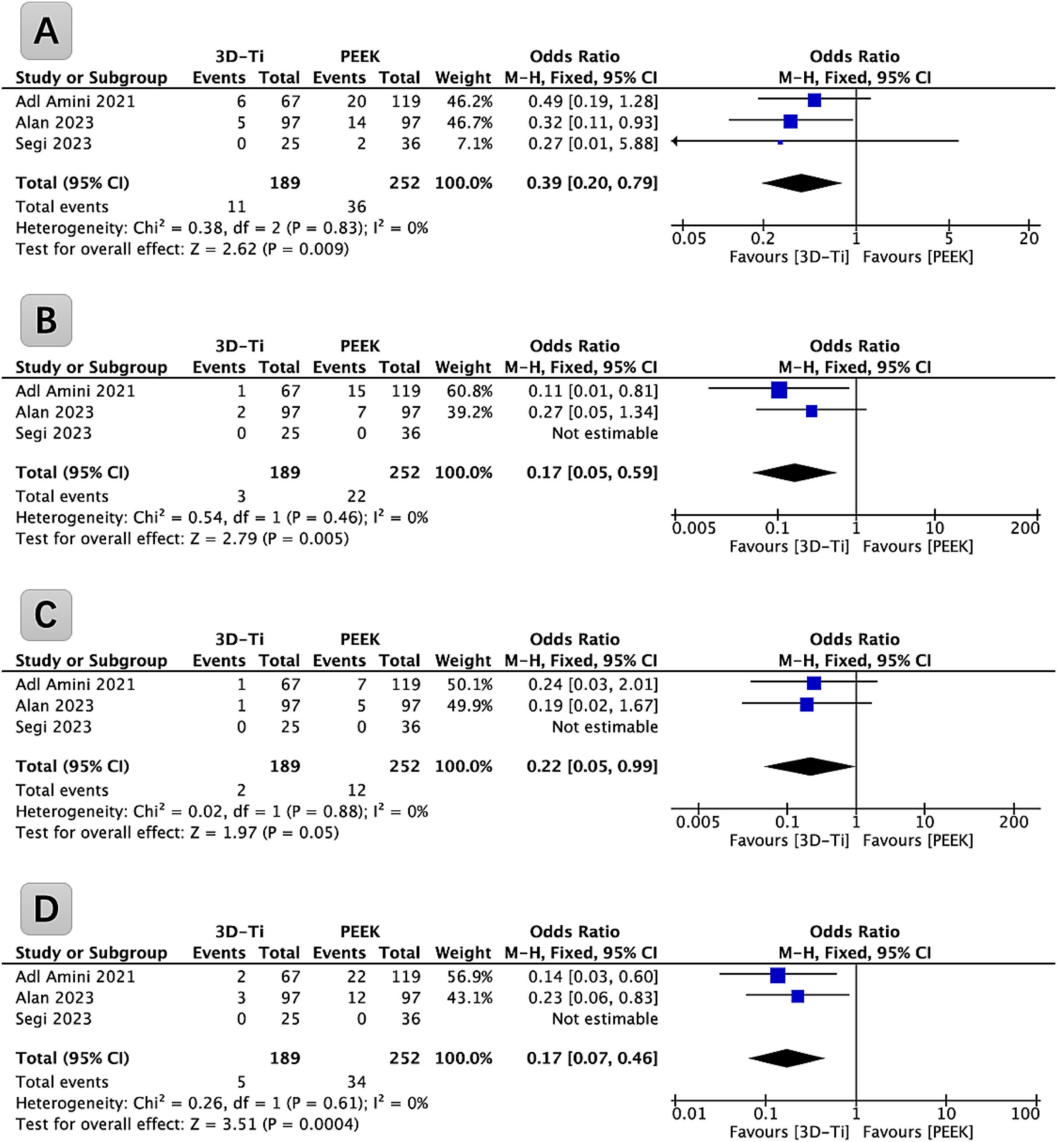
**(A)** Forest plot of 3D-Ti group compared with PEEK group in terms of Marchi Grade I subsidence in LLIF; **(B)** Forest plot of 3D-Ti group compared with PEEK group in terms of Marchi Grade II subsidence in LLIF. **(C)** Forest plot of 3D-Ti group compared with PEEK group in terms of Marchi Grade III subsidence in LLIF. **(D)** Forest plot of 3D-Ti group compared with PEEK group in terms of severe subsidence in LLIF. LLIF, lumbar lateral interbody fusion, 3D-Ti, 3D-printed porous titanium, PEEK, polyetheretherketone.

Among the identified studies, three provided data relevant to the subsidence of grade I cages, as defined by the Marchi grade classification (Grade 0 denotes a postoperative disc height loss ranging from 0 to 24%; Grade I means a subsidence within the range of 25 to 49%; Grade II encompasses a subsidence extent of 50 to 74%; and Grade III designates a subsidence involving a 75 to 100% loss of postoperative disc height), and were incorporated into a comprehensive analysis (Figure 3A). These studies collectively covered a cohort of 265 patients (441 levels) who underwent LLIF surgery. Statistical analysis revealed a significantly lower incidence of grade I cage subsidence in the 3D-Ti cage group compared to the PEEK group (*p* = 0.009, OR = 0.39; 95% CI: 0.20 to 0.79). In particular, the grade I cage subsidence rate in the 3D-Ti group was 5.82%, while the PEEK group exhibited a rate of 14.29%.

Among the identified studies, three provided data (265 patients [441 levels]) relevant to grade II cage subsidence, as defined by the Marchi classification, and were incorporated into a comprehensive analysis (Figure 3B). Statistical analysis revealed a significantly lower incidence of grade II cage subsidence in the 3D-Ti cage group compared to the PEEK group (*p* = 0.005, OR = 0.17; 95% CI: 0.05 to 0.59). In particular, the grade II cage subsidence rate in the 3D-Ti group was 1.59%, while the PEEK group exhibited a rate of 8.73%.

Among the identified studies, three provided data (265 patients [441 levels]) relevant to grade III cage subsidence, as defined by the Marchi classification, and were incorporated into a comprehensive analysis (Figure 3C). Statistical analysis revealed a significantly lower incidence of grade III cage subsidence in the 3D-Ti cage group compared to the PEEK group (*p* = 0.05, OR = 0.22; 95% CI: 0.05 to 0.99). In particular, the severe cage subsidence rate in the 3D-Ti group was 1.06%, while the PEEK group exhibited a rate of 4.76%.

The analysis was carried out based on the severe cage subsidence defined by the Marchi classification (grades II and III), as shown in Figure 3D. Statistical analysis revealed a significantly lower incidence of severe cage subsidence in the 3D-Ti cage group compared to the PEEK group (*p* = 0.0004, OR = 0.17; 95% CI: 0.07 to 0.46). In particular, the severe cage subsidence rate in the 3D-Ti group was 2.64%, while the PEEK group exhibited a rate of 13.49%.

### 3.4 Quality analysis and publication bias

The robustness of the synthesized evidence was bolstered by the high quality of included studies. As shown in Table 3, all studies exceeded predetermined quality thresholds, evidenced by NOS scores of 5 or more stars. This underscores the reliability of the findings reported in this meta-analysis.

**Table 3:** Quality assessment of the included studies.

Studies	Selection				Comparability	Exposure			Total scores (of 9)
	Is the case definition adequate?	Representativeness of the cases	Selection of controls	Definition of controls	Comparability of cases and controls on the basis of the design or analysis	Ascertainment of exposure	Same method of ascertainment for cases and controls	Non-response rate	
Adl Amini 2021 (25)	☆	☆			☆	☆	☆	☆	7
Alan 2023 (26)	☆	☆			☆	☆	☆		6
Segi 2023 (27)	☆	☆	☆		☆	☆	☆		7

**Figure 4:**
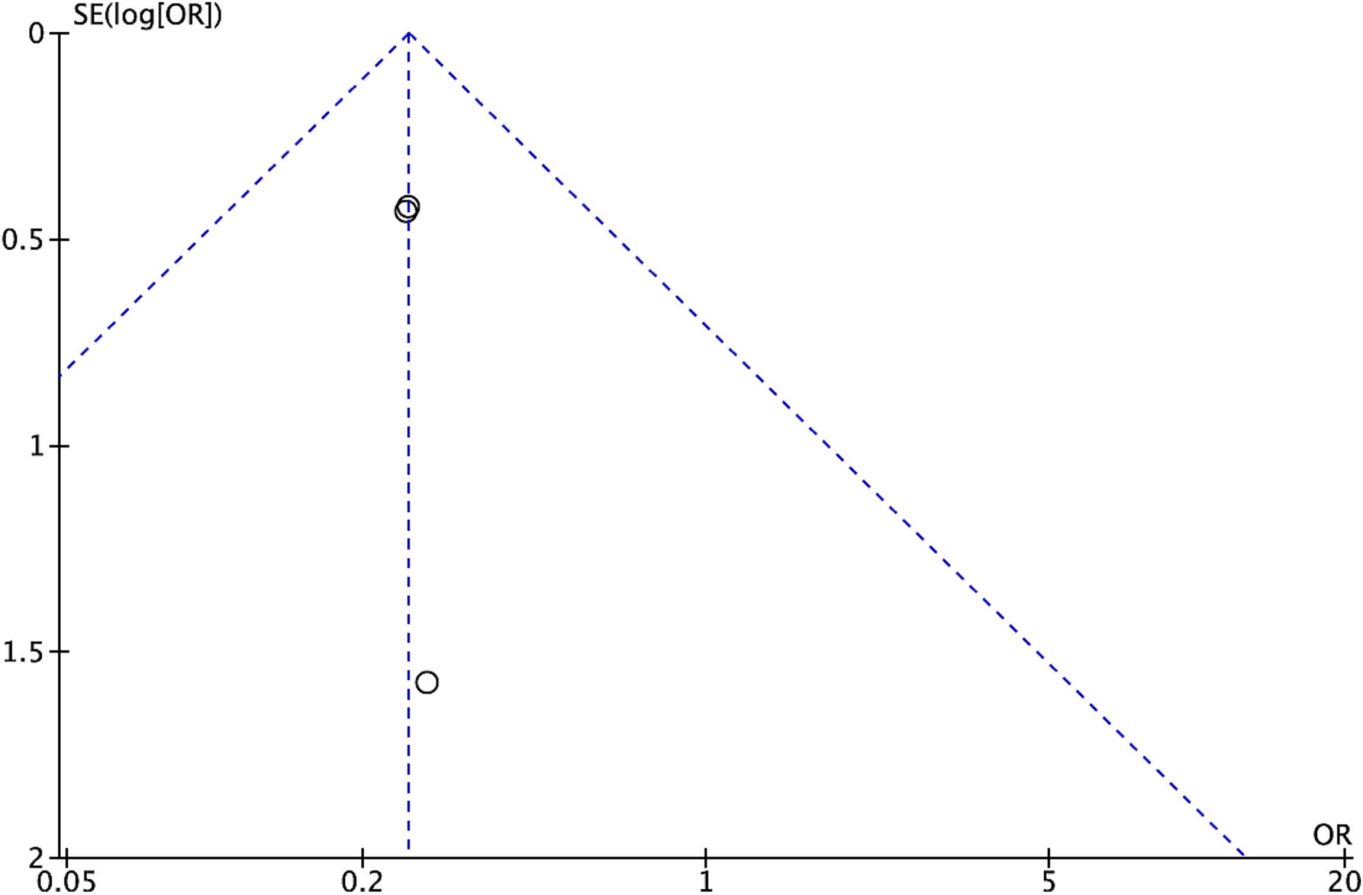
Funnel plot of publication bias for cage subsidence.

Further investigation of possible publication bias, particularly related to general comorbidity, was conducted by visual inspection of a funnel plot (Figure 4). The observed symmetrical distribution within the plot suggests a low risk of publication bias influencing the results.

Finally, the GRADE method was used in Table 4 to systematically assess confidence in the overall findings.

**Table 4:** A credibility assessment according to the GRADE scoring system.

Outcome	No. of participants (studies)	Quality of the evidence (GRADE)	Comments and overall results
Cage subsidence (Marchi Grade I, II and III)^#^	265 (3)	Moderate	Statistical analysis revealed a significantly lower incidence of cage subsidence in the 3D-Ti cage group compared to the PEEK cage group (*p* < 0.00001)
Marchi Grade I^#^	265 (3)	Moderate	Statistical analysis revealed a significantly lower incidence of grade I cage subsidence in the 3D-Ti cage group compared to the PEEK group (*p* = 0.009)
Marchi Grade II^#^	265 (3)	Moderate	Statistical analysis revealed a significantly lower incidence of grade II cage subsidence in the 3D-Ti cage group compared to the PEEK group (*p* = 0.005)
Marchi Grade III^#^	265 (3)	Moderate	Statistical analysis revealed a significantly lower incidence of grade III cage subsidence in the 3D-Ti cage group compared to the PEEK group (*p* = 0.05)
Severe cage subsidence (Marchi Grade II and III)^#^	265 (3)	Moderate	Statistical analysis revealed a significantly lower incidence of severe cage subsidence in the 3D-Ti cage group compared to the PEEK group (*p* = 0.0004)

3D-Ti, 3D-printed porous titanium; PEEK, polyetheretherketone. #, Marchi Grade I indicates a subsidence within the range of 25 to 49% of postoperative disc height; Grade II corresponds to a subsidence extent of 50 to 74%; and Grade III designates a subsidence involving a 75 to 100% loss of postoperative disc height.

## 4 Discussion

To our knowledge, this study is the first to compare the subsidence rates of 3D-Ti cages and PEEK cages after LLIF. Our results found a statistically significant reduction in the overall subsidence rate of 3D-Ti cages compared to PEEK cages in LLIF. According to the Marchi grading, severe subsidence is also rarer in 3D-Ti groups than in PEEK groups after LLIF. Previous studies have compared titanium and PEEK cages after cervical and lumbar fusion, but their results have varied considerably. A meta-analysis (28) (included five studies) found that titanium cages had significantly higher subsidence rates compared to PEEK cages after cervical and lumbar fusion surgery (OR 3.59, *p* = 0.015). However, in a recent meta-analysis published by Tan et al. (29) in 2021 (included seven studies), it was found that after the cervical and lumbar fusion procedure, patients implanted with titanium cages (*n* = 259) exhibited a higher rate of cage subsidence (RR 2.17, *p* = 0.02) compared to those implanted with PEEK cages (*n* = 231). However, the specific surgical procedures for fusion were not explicitly detailed in the literature. Furthermore, another meta-analysis (30) (included four studies) found that the range of subsidence rates after cervical and lumbar fusion was 0–36% in the titanium group and 0–31% in the PEEK group, without statistically significant differences in their subsidence rates (OR 0.91, *p* = 0.71).

LLIF is a minimally invasive surgical procedure designed to treat a variety of spinal conditions, including degenerative disc disease, spondylolisthesis, and adult scoliosis (1). This procedure stabilizes the affected spinal segment by inserting a large interbody cage through a lateral approach into the disc space. Compared to the traditional posterior approach, LLIF reduces damage to the posterior tissues, thereby facilitating a faster recovery time (3). However, LLIF is indicated primarily for the treatment of patients with degenerative lumbar spine disease, who often experience a high incidence of subsidence of fusion due to advanced age and underlying conditions such as osteoporosis and endplate inflammation (25). The subsidence of the cage alters the mechanical stability of the spine, which can lead to recurrent pain, neurological deficits, impaired fusion, the need for revision surgery, and accelerated degeneration of adjacent segments (31). Consequently, subsidence is a significant complication after LLIF. Cage subsidence is operationally defined as the displacement of the implant within the upper and lower endplates of adjacent vertebrae (32). Although the occurrence of subsidence after interbody fusion is prevalent in lumbar spine surgeries, a consensus on its etiology and clinical significance remains elusive. In particular, a certain degree of subsidence can be clinically asymptomatic; however, excessive subsidence poses the risk of mechanical failure within the anterior lumbar column, resulting in bone failure, loss of height in the intervertebral, and sagittal imbalance (33). To systematically analyze cage subsidence, we employed the Marchi grading system, stratifying it into I, II, and III grading. Our findings reveal a noteworthy observation: the 3D-Ti group exhibited a significantly lower subsidence rate across all classified levels compared to the PEEK group. In response to the problem of cage subsidence that occurs during lumbar interbody fusion surgery, several possible explanations are provided below. First, the inherent challenge arises from excessive intervertebral pressure and localized stress concentration, stemming from the need to support the intervertebral space adequately during the placement of the PEEK cage. This necessity often results in the selection of a cage height that exceeds the intervertebral space, leading to increased intervertebral pressure. Furthermore, mismatches between the ends of the PEEK cage and the upper and lower endplates of the vertebral body contribute to a decrease in contact area and a subsequent stress concentration. A contrasting approach is exemplified by the 3D-Ti cage, which allows for individualized customization. This personalized shaping conforms more closely to the diseased intervertebral space, effectively mitigating intervertebral pressure, minimizing stress concentration, and consequently reducing the likelihood of cage subsidence. In addition, PEEK cage implementations incorporate serrated structures embedded in the bone surface to prevent fusion displacement. However, this approach introduces a degree of damage to the vertebral endplates, increasing the susceptibility to cage subsidence (32). Conversely, the roughened surface of the 3D-Ti cage serves to enhance friction between the fusion device and the endplate, thereby impeding cage subsidence (34). Furthermore, the 3D-Ti cage introduces an innovative porous structure, strategically designed to align its elastic modulus more closely with that of the human vertebral body. This design feature effectively diminishes the effect of stress masking on the contact surface between the fusion device and the vertebral body, decelerating the loss of vertebral bone volume and, consequently, mitigating the subsidence of the vertebral body. The incorporation of a porous structure represents a creative solution to enhance biomechanical compatibility and prevent undesirable outcomes associated with interbody fusion procedures (35).

The clinical implications of our findings suggest that the reduced subsidence rate associated with 3D-Ti cages implies better mechanical stability post-surgery, leading to fewer complications and potentially fewer revision surgeries. The stability provided by 3D-Ti cages improves the conditions for successful spinal fusion, with better osseointegration that promotes robust and faster fusion processes. Patients with 3D-Ti cages are likely to experience fewer recurrent pain and neurological deficits, resulting in better overall outcomes and smoother postoperative recovery. They are also expected to have improved long-term spinal stability and alignment, reducing the progression of adjacent segment degeneration and improving overall spinal health, satisfaction, and quality of life. Given these findings, 3D-Ti cages may be preferred over PEEK cages, especially for patients at higher risk of subsidence due to factors such as osteoporosis or poor bone quality. Surgeons may consider patient-specific factors such as age, bone density, and activity level when selecting cage materials, with older patients or those with compromised bone quality potentially benefiting more from 3D-Ti cages. Surgeons might also incorporate the likelihood of subsidence into their preoperative planning and postoperative monitoring protocols, focusing on fusion progression.

### 4.1 Strength and limitations

This meta-analysis adheres to the guidelines established by the PRISMA and represents the inaugural scholarly effort in this domain, systematically comparing the subsidence rates of 3D-Ti and PEEK cages in LLIF through a meticulous exploration of the existing literature. However, certain limitations merit consideration. First, the incorporated studies exhibited a modest sample size, with a notable absence of prospective and RCT designs. Second, the omission of key factors, such as smoking, osteoporosis, bone mineral density, and differ in the use of posterior instrumentation in some studies detracts from the comprehensive evaluation of pertinent variables. Third, the absence of reporting on the specifics regarding the type and size of intervertebral cages utilized in certain studies introduces ambiguity. Fourth, Lastly, the paucity of various outcomes, including clinical measurements, fusion rates, and other pertinent metrics, represents a notable gap in the comprehensive assessment of the LLIF outcomes.

## 5 Conclusion

This study is the first to compare the subsidence rates of 3D-Ti cages and PEEK cages after LLIF. Our results demonstrate a statistically significant reduction in the overall subsidence rate of 3D-Ti cages compared to PEEK cages in LLIF. According to the Marchi grading, severe subsidence is also rarer in the 3D-Ti group than in the PEEK group after LLIF.

## Data Availability

The raw data supporting the conclusions of this article will be made available by the authors, without undue reservation.
