# Corrigendum: 3D-printed porous titanium versus polyetheretherketone cages in lateral lumbar interbody fusion: a systematic review and meta-analysis of subsidence

**DOI:** 10.3389/fmed.2025.1594590

**Published:** 2025-04-10

**Authors:** Shu-Xin Liu, Teng-Hui Zeng, Chien-Min Chen, Li-Ru He, An-Ping Feng, Shang-Wun Jhang, Guang-Xun Lin

**Affiliations:** ^1^Department of Orthopedics, Panjin Central Hospital, Panjin, Liaoning, China; ^2^Department of Orthopedics, The Second People's Hospital of Shenzhen (The First Affiliated Hospital of Shenzhen University), Shenzhen, Guangdong, China; ^3^Division of Neurosurgery, Department of Surgery, Changhua Christian Hospital, Changhua, Taiwan; ^4^Department of Leisure Industry Management, National Chin-Yi University of Technology, Taichung, Taiwan; ^5^Department of Biomedical Sciences, National Chung Cheng University, Chiayi, Taiwan; ^6^Department of Anesthesia and Surgery, The First Affiliated Hospital of Xiamen University, Xiamen University, Xiamen, Fujian, China; ^7^Department of Orthopedics and Traumatology of Traditional Chinese Medicine, The Third Hospital of Xiamen, Xiamen, China; ^8^Department of Orthopedics, The First Affiliated Hospital of Xiamen University, School of Medicine, Xiamen University, Xiamen, China; ^9^The School of Clinical Medicine, Fujian Medical University, Fuzhou, Fujian, China

**Keywords:** lateral lumbar interbody fusion, polyetheretherketone, interbody cage, titanium, subsidence

In the published article, there was an error in affiliation(s) [8, 9]. Instead of “^8^Department of Orthopedics, Tongren Hospital, Shanghai Jiao Tong University School of Medicine, Shanghai, China; ^9^Department of Orthopedics, The First Affiliated Hospital of Xiamen University, School of Medicine, Xiamen University, Xiamen, China,” it should be “^8^Department of Orthopedics, The First Affiliated Hospital of Xiamen University, School of Medicine, Xiamen University, Xiamen, China; ^9^ The School of Clinical Medicine, Fujian Medical University, Fuzhou, Fujian, China.”

The authors apologize for this error and state that this does not change the scientific conclusions of the article in any way. The original article has been updated.

